# Presence of embryonic DNA in culture medium

**DOI:** 10.18632/oncotarget.18852

**Published:** 2017-06-29

**Authors:** Linlin Yang, Qiaoying Lv, Wei Chen, Jian Sun, Yu Wu, Yiying Wang, Xiong Chen, Xiaojun Chen, Zhenbo Zhang

**Affiliations:** ^1^ The Reproductive Medicine Center of Department of Obstetrics and Gynecology, Shanghai General Hospital, Shanghai Jiao Tong University, Shanghai 200080, China; ^2^ Department of Obstetrics and Gynecology, Shanghai First People’s Hospital, Baoshan Branch, Shanghai 201900, China; ^3^ Obstetrics and Gynecology Hospital of Fudan University, Shanghai 200011, China; ^4^ Department of Obstetrics and Gynecology, Henan Province People’s Hospital, Zhengzhou 450000, China

**Keywords:** embryonic culture medium, intracytoplasmic sperm injection, polymerase chain reaction, preimplantation genetic diagnosis, in vitro fertilization

## Abstract

Preimplantation genetic diagnosis (PGD) has successfully assisted couples with genetic diseases to conceive healthy babies during the past decades. However, biopsy of the blastomere has potential lesion to the embryos which commonly results in abortion. Thus, a noninvasive PGD is needed. In the past, the presence of genetic materials in maternal plasma or serum has triggered a great innovation of noninvasive prenatal diagnosis. Nevertheless, it is not clear whether embryonic DNA is also present in embryonic culture medium. Here, a rapid-boiling method has been used to harvest DNA from the medium or the discarded embryos, following Polymerase Chain Reaction (PCR) was applied to detect the dissociative DNA by amplifying SRY gene (Y-chromosome). For the first time, the Y sequences were detected in the medium which were used to culture embryo for above 3 days. None of the positive signal was examined in Day 1 and Day 2 embryonic culture medium. Our findings suggest that the Y chromosome fragments from the embryo may release into its culture medium. If validated in a larger cohort, detection of SRY gene may prove to be a useful method to screen Y-linked genetic disease. More importantly, detecting the free DNA in the embryonic culture medium may represent a novel strategy for noninvasive PGD.

## INTRODUCTION

Infertility seriously impairs mature couples’ reproductive capacity, leading to an increasing need for the use of assisted reproductive technology (ART) and *In vitro* fertilization (IVF). IVF could provide individual approaches to the patients suffered infertility according to their different infertility problems, such as PGD, single embryo transfer, blastocyst transfer and so forth.

The most important goal of reproductive medicine is to select a healthy embryo to aid infertility couples to get healthy babies. In 1990, Handyside AH successfully prevented X-linked genetic disease passing on to the next generation for the first time through sex identification of the fetus by amplifying Y chromosome-specific repeat sequence of a single cell at cleavage stage [1]. From then on, human IVF technology combined with PGD has succeeded in increasing the likelihood of having a healthy fetus [2]. Routinely, PGD can be carried out by direct genetic assessment by removing a single or multiple cells from preimplantation embryos in either cleavage stage [3] or blastocyst stage [4]. Although blastomere biopsy does directly assess the genome DNA of *in vitro* fertilized embryos, this invasive procedure is more likely to result in the serious damage of IVF units [5]. Besides, genetic diagnosis in this stage is not so reliable especially in embryos with mosaicism [6]. In contrast, trophectoderm biopsy by removing more than one cells for genetic diagnosis is more reliable because it is more representative of the complete chromosomal complement of the preimplantation embryos [7]. However, biopsy based on embryonic cells is still an invasive operation although this technique has been worldwide used in IVF Units. Thus, it is necessary to seek for a noninvasive technology to screen embryos for inherited diseases or aneuploidy.

In 1997, Lo YMD and his colleagues reported the presence of cell-free fetal DNA in maternal plasma for the first time, which has offered a window for noninvasive prenatal testing (NIPT) [8]. Based on this concept, researchers showed an increasing interesting to explore whether it also exists embryo-origin free DNA in embryonic culture medium. S Palini stated that genomic DNA was present in about 90% of blastocyst fluid samples harvested during the vitrification procedure [9]. However, most IVF Units select Day 3 fresh embryo for transfer, not the blastocyst. Thus, in this condition the blastocyst fluid is not available. This prompted us to investigate whether genomic DNA can be detected in the medium at embryo culture different stages. Recently, it was demonstrated a medium-based PGD test for α-thalassemias^−SEA^ by comparing single embryo cell biopsy with the spent culture medium. The diagnosis efficiency of medium-based α-thalassemias^−SEA^ detection significantly increased when compared with that of embryo cell biopsy by fluorescent gap PCR analysis [10]. On the other hand, S. Stigliani et al. reported that the amount of DNA is larger in embryos with bad quality cleavage when compared with high-grade embryos and a high mitochondrial DNA (mtDNA)/genomic DNA (gDNA) ratio in spent medium was associated with successful implantation outcome [11, 12]. In this regard, DNA content in the spent medium, in combination with morphological grading, can predict the blastocyst potential and implantation outcome. The discovery of DNA in embryonic culture medium provides a novel strategy for future noninvasive PGD technology.

In this study, we attempted to investigate the presence of genomic DNA by amplifying SRY genes (Y chromosome) from Day 1 to Day 6 in spent medium and embryos using PCR-based approach. For the first time, we detected the gene located in Y-chromosome in the spent morula and blastocyst culture medium. This finding will provide a novel noninvasive technology for screening or detecting embryo DNA from embryonic culture medium instead of embryo itself.

## RESULTS

The SRY gene was successfully amplified in Day 3 to Day 6 culture medium, the 189bp amplified product by PCR was obviously observed. None of the signal was detected in Day 1 and Day 2 culture medium. Y-negative signals were detected in the reaction system used water, G1 and G2 medium as templates, respectively. Whereas sperm sample showed positive signal when it served as a positive loading control. This implies that embryonic culture medium contains the genetic materials.

To further determine if these DNA originate from corresponding embryo, the paired embryo and medium were used to amplify the Y-chromosomal sequences. As shown in Table 1, five Day 1-medium samples from three cases and four Day 2-medium samples from two cases showed no Y-positive signals after Y-PCR, nevertheless, two of four Day 2-embryo had positive Y signals paralleled with each matched culture medium. Day 1-embryo has not been enrolled in this study because the spent embryos are not available in clinical practice. These results suggest that none of the DNA or its fragments release into the culture medium at the beginning of two days in embryo development. Nine paired Day 3-medium and embryo were harvested from six cases, four paired showed positive Y-PCR signals, five negative signals, which implies that four embryos were males, five were females. This suggests that the sex can be simultaneously detected in embryo and its corresponding medium. Similar patterns also were observed in Day 4 to Day 6 paired embryo and medium. It is commonly acknowledged that the genetic materials in culture medium come from embryo. The lazer assistant hatching was performed to release the blastocyst fluid into culture medium, subsequently, the medium was harvested to amplify the Y-sequences. As we expected, enrolled three paired Day 6-blastocysts and medium showed stronger positive Y-PCR signal (Table 1).

**Table 1:** Amplification of embryonic Y-chromosomal sequences from culture medium

Embryo no.	D1		D2		D3		D4		D5		D6		D6 (H)	
	CM	E	CM	E	CM	E	CM	E	CM	E	CM	E	CM	E
1	—	NC	—	—	+	+	+	+	+	+	—	—	+	+
2	—	NC	—	+	—	—	—	—	+	+	—	—	+	+
3	—	NC	—	+	—	—	+	+	+	+	—	—	+	+
4	—	NC	—	—	+	+	+	+	—	—	+	+		
5	—	NC			+	+								
6					+	+								
7					—	—								
8					—	—								
9					—	—								

The paired embryo and medium from Day 1 to Day 6. CM=culture medium; E=embryo; H=hatching; NC=not collected.

## DISCUSSION

In this study, we have detected the Y-chromosome in the spent culture media and discarded embryo for the first time. This finding demonstrates that embryonic culture media contains DNA fragment originating from corresponding morula or blastocyst, which approves a possibility to screen the gene profiles by detecting the medium. It also provides a promising strategy for noninvasive PGD.

**Figure 1:**
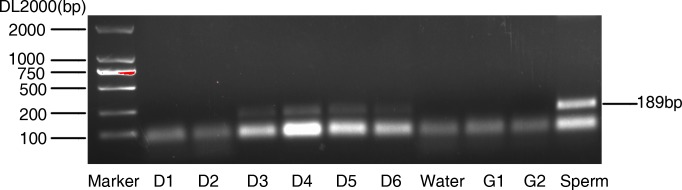
Amplification of Y-chromosomal sequences from blastocyst culture medium The culture medium collected from the indicated time. Water, G1 and G2 culture medium used as negative control, sperm served as positive control.

Currently, the main method to assess the embryo quality is scoring the embryo by using morphological factors, including blastomeric or embryonic size, the symmetry and rate of embryo cleavage, cumulus-coronal cell morphology, cytoplasm granularity and embryo polarity. Nonetheless, morphological features cannot represent the embryo genomic DNA status. Commonly, in clinical practice, some of the embryos with perfect morphology resulted in unsuccessful pregnancy, even spontaneous abortion after transfer operation. This may attribute to DNA mutation, chromosome inversion, translocation or deletion. Therefore, prior to embryo implantation, DNA assay is necessary and required, instead of only depending on its morphology. In the present study, the released DNA was detected from Day 3 to Day 6 culture medium (Figure 1), not in Day 1 and Day 2. This maybe reasonable because fertilization and first cleavage normally happened in the first two days, no more DNA fragments accumulated. DNA fragments increased with the culture and cleavage times. So the genetic materials can be easily detected in blastocyst stage.

In current study, we used a rapid-boiling method to treat samples including culture medium and embryonic samples. The main reason is that it is hard to extract the DNA from the 12.5ul culture medium or four cell- and eight cell-embryo, so the rapid-boiling method could lead to the DNA release as soon as possible. In spite of less DNA, we still obtained the Y-PCR signal when we used all the culture medium or all embryonic lysis. As shown in Table 1, some of the paired culture medium and embryo were negative Y-PCR signal. This result is reasonable because the embryo may represent a female, there is no Y chromosome fragments released into culture medium. So Y-PCR positive signal is not possible to present in the corresponding medium. Inversely, paired medium and embryo with Y-PCR positive signals might be males.

The indeed merit to detect the SRY is to point out a novel strategy for noninvasive PGD. Current PGD is biopsy-dependent, which has the potential risk to impair embryo. Our finding is not limited to detect the sex-linked disorders, which indicates that the embryonic culture medium contains the embryonic genetic information. In this regard, the genetic material in embryonic culture medium could be also used to detect other genetic diseases before embryo transfer. Therefore, the high qualified and healthy embryos can be selected for transfer by a noninvasive method.

It is possible that the embryonic culture medium could be used for noninvasive PGD, However, in a traditional way the DNA contamination from other unfused sperm may limit its application. In current study, all the cases were performed by ICSI, which can avoid contamination from surrounding circumstances.

Culture medium PCR is an inexpensive, noninvasive and harmless technique in screening inherited genetic diseases. Although this method is not so accurate due to false positive, it can be acted as a primary screening for inherited genetic diseases. On the other hand, we supposed that current PGD techniques including next generation sequencing and microarray, along with embryonic culture medium, might be the novel strategies in future reproductive medicine.

## MATERIALS AND METHODS

### Ethical approval

All the clinical information was obtained from the Reproductive Medicine Center, Shanghai General Hospital, Shanghai Jiao Tong University and the clinical analysis was approved by the Ethics Committee of Shanghai Jiao Tong University School of Medicine.

### Patients and intracytoplasmic sperm injection (ICSI) procedure

From March 2015 to October 2016, patients who visited the reproductive clinic of our hospital for infertility and sought for the help of ART and IVF were prospectively recruited. These patients using ovulation stimulants underwent transvaginal oocyte retrieval and fertilization using ICSI as previously described [13]. Briefly, Transvaginal sonography (TVS) guided follicular puncture was processed 36 hours after the hCG (Mochida Pharmaceutical) injection. The harvested oocytes were denudated enzymatically with recombinant human hyaluronidase and then pipetted mechanically with commercial glass pipettes. The retrieved normal morphological spermatozoa were injected into the MII oocytes.

### Sample collection and preparation

After ICSI, all fertilized oocytes were cultured in a humidified chamber at 37°C with 6% CO_2_ and 5% O_2_ from Day 1 to Day 6 respectively. There were five Day 1-medium samples, four paired embryos and its medium in Day 2, nine paired embryos and its medium in Day 3, four paired embryos and its medium in Day 4, five paired embryos and its medium in Day 5, four paired embryos and its medium in Day 6. Embryos were cultured in G1 (G1 plus; VitriLife) medium (12.5ul) among the first three days and G2 (G2 plus; VitriLife) medium (12.5ul) in the latter period. 10ul culture medium and matched embryos were harvested respectively. The sample preparation was described previously [8]. Briefly, the samples were incubated at 56°C for 30 min on a heat block, then maintained at 100°C for 20 min. The treated samples were stocked at 4°C until PCR. During the procedure of culture medium preparation and embryos collection, all operators wore gloves, masks, caps and lab coats to assure compliance with sterile principles.

### PCR assay

The primers of SRY gene were: 5’-CATCCAGAGCGTCCCTGGCTT -3’(Forward Primer); 5’-CTTTCCACAGCCACATTTGTC -3’(Reverse Primer). The amplification conditions for SRY were 95°C for 2 min and 40 cycles of 95°C for 5 s and 60°C for 34 s according to the manufacturer's protocol (Takara). The PCR products were visualized in electrophoresis on 2% agarose gels with Gel-Red (Bitium) staining by GelDoc XR Image analyzer (Bio-Rad) as depicted before [14]. Water/G1/G2 and sperm were used as negative and positive templates, respectively. Each experiment repeats three times.
